# Simplified Method for *Agrobacterium*-Mediated Genetic Transformation of *Populus* x *berolinensis* K. Koch

**DOI:** 10.3390/mps7010012

**Published:** 2024-01-26

**Authors:** Vasiliy V. Pavlichenko, Marina V. Protopopova

**Affiliations:** Siberian Institute of Plant Physiology and Biochemistry, Siberian Branch of the Russian Academy of Sciences, Lermontov St., 132, Irkutsk 664033, Russia

**Keywords:** *Agrobacterium*-mediated plant transformation, micropropagation, *Populus × berolinensis*, transgenic plants, woody plants, poplar

## Abstract

The rapid advancement of genetic technologies has made it possible to modify various plants through both genetic transformation and gene editing techniques. Poplar, with its rapid *in vitro* growth and regeneration enabling high rates of micropropagation, has emerged as a model system for the genetic transformation of woody plants. In this study, *Populus × berolinensis* K. Koch. (Berlin poplar) was chosen as the model organism due to its narrow leaves and spindle-shaped crown, which make it highly suitable for *in vitro* manipulations. Various protocols for the *Agrobacterium*-mediated transformation of poplar species have been developed to date. However, the genetic transformation procedures are often constrained by the complexity of the nutrient media used for plant regeneration and growth, which could potentially be simplified. Our study presents a cheaper, simplified, and relatively fast protocol for the *Agrobacterium*-mediated transformation of Berlin poplar. The protocol involved using internode sections without axillary buds as explants, which were co-cultivated in 10 µL droplets of bacterial suspension directly on the surface of a solid agar-based medium without rinsing and sterile paper drying after inoculation. We used only one regeneration Murashige and Skoogbased medium supplemented with BA (0.2 mg·L^−1^), TDZ (0.02 mg·L^−1^), and NAA (0.01 mg·L^−1^). Acetosyringone was not used as an induction agent for *vir* genes during the genetic transformation. Applying our protocol and using the binary plasmid pBI121 carrying the *nptII* selective and *uidA* reporter genes, we obtained the six transgenic lines of poplar. Transgenesis was confirmed through a PCR-based screening of kanamycin-selected regenerants for the presence of both mentioned genes, Sanger sequencing, and tests for detecting the maintained activity of both genes. The transformation efficiency, considering the 100 explants taken originally, was 6%.

## 1. Introduction

It has been over 35 years since the first successful *Agrobacterium*-mediated genetic transformation of poplar was demonstrated [1]. Since then, scientists from around the world have developed, improved, or adapted numerous protocols for the regeneration of transgenic plants after their *Agrobacterium*-mediated transformation. The main parameters being improved in the transformation protocols are the regeneration time and the number and composition of various nutrient media used during the regenerants’ selection. The fewer different types of media used, the simpler they are, the faster and cheaper it is possible to achieve the desired result. Poplars are known for being one of the fastest-growing temperate trees and woody plants from *in vitro* cultures [2,3] and they emerge as a model for woody plants [4]. Therefore, the development of a rapid, cheap, and easy protocol for the genetic transformation of poplar is an important task in the genetic engineering of woody plants. Various poplar plant parts including leaves, petioles, internode segments, and roots, can be used as explants for *Agrobacterium*-mediated transformation [2,5,6,7,8,9]. The regeneration of plants can be carried out through the callus or direct morphogenesis, and all types of explants are suitable for both. Leaves are the most abundant and easily accessible organs in poplar, but they are difficult to slice and handle, and require high concentrations of hormones to induce a callus or organogenesis. In addition, the leaves wither quickly when sliced before co-cultivation with *Agrobacterium*. The roots of *in vitro* plants are very fragile, dry very quickly during cutting, and are difficult to handle during co-cultivation with *Agrobacterium* suspension and further manipulations during plant regeneration. Leaf petioles are also a convenient explant, but they are too small to be obtained from one *in vitro* plant (only 5–8 pcs) in sufficient quantities for transformation and require high concentrations of hormones for organogenesis induction. An alternative source for plants regeneration is a callus. However, calli have some disadvantages, such as the need for constant maintenance in laboratory conditions, along with the *in vitro* poplar plants, as well as the possible appearance of chimeric plants after *Agrobacterium*-mediated transformation [10]. In our opinion, the most optimal *in vitro* explant type for *Agrobacterium*-mediated poplar transformation, in terms of quantity, quality, and simplicity, is internode sections without axillary buds. They can be obtained from one *in vitro* plant in 10 to 15 pieces. Lower concentrations of hormones are required for the regeneration of new plants from internode sections, making it possible to reduce the cost of the poplar genetic transformation process. Direct morphogenesis, which can be induced in the internode sections, both simplifies the transformation procedure and reduces the risk of chimeric plants compared to the callus pathway.

In this paper, we present a simplified procedure for obtaining transgenic Berlin poplar using internode sections as explants.

## 2. Materials and Methods

### 2.1. Nutrient Media Composition

Nutrient media were prepared on the basis of commercial Murashige and Skoog Basal Salt Mixture (MS) (MS5524, Sigma–Aldrich, Saint Louis, MO, USA). Media for co-cultivation, regeneration, shoot elongation, and rooting were prepared by supplementing the base MS5524 medium with the components listed in Table 1.

The freshly prepared medium was autoclaved at 121 °C and 220 kPa for 15 min and dispensed in 50 mL portions into sterile polycarbonate GA-7-3 culture vessels (Sigma-Aldrich, Saint Louis, MO, USA) and in 10 mL portions into 150 × 25 mm glass tissue-culture tubes (Z681784, Sigma-Aldrich, Saint Louis, MO, USA) with plastic vented closures. Kanamycin sulfate (A1493, AppliChem, Barcelona, Spain) and cefotaxime (Lekko, Volginskiy setl., Russia) were added to the air-cooled autoclaved medium (55 °C) to prevent thermal degradation of the antibiotic.

### 2.2. Micropropagation Technique and Growing Conditions

**Figure 1:**
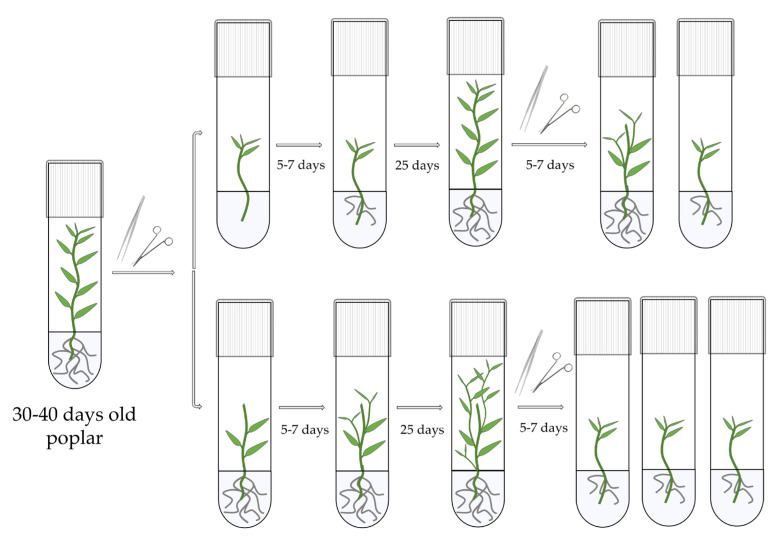
Scheme of the *Populus × berolinensis* micropropagation through sequential cutting and rooting of the apical shoot parts.

The present study utilized *Populus × berolinensis* K. Koch, also known as Berlin poplar, as the model object. Long-term culture of *Populus × berolinensis* is part of the woody plants *in vitro* collection at the Siberian Institute of Plant Physiology and Biochemistry (Irkutsk, Russia). Berlin poplar is a convenient species for studying woody plants, including the applications of genetic engineering methods, due to its relatively fast growth (30–40 days from rooting *in vitro* to a 10 cm plant), high rooting efficiency (close to 100%) during *in vitro* propagation, narrow leaves that occupy little space in culture tubes, and easy regeneration from roots and internodal segments. The *in vitro* propagation of poplar was achieved by sequential cutting and rooting the plant’s apical shoot parts, with simultaneous replantation of the rooted stump in fresh nutrient medium supplemented by 0.15 mg·L^−1^ of IBA (Figure 1, Table 1).

By using this approach, it is possible to obtain 4–5 rooted poplars from one plant within 35–40 days. The plants were grown *in vitro* at 24 °C in an air-conditioned room with a 16/8 h day/night photoperiod, illuminated by TL5 HE 28W/865 fluorescent lamps (BMC, Guzhen, China) at 5000 Lux [11]. The ambient humidity was not controlled.

### 2.3. Agrobacterium-Mediated Transformation Protocol

**Figure 2:**
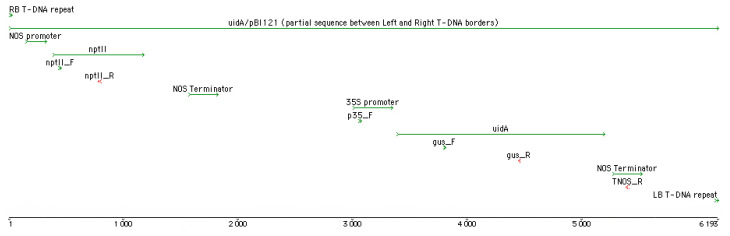
Schematic representation of the key elements and primer positions on the T-DNA region of *uidA*/pBI121, constructed using GenBank sequence (ID: AF485783.1) in Sequencher 5.1 software. The arrow colors indicate forward (green) or reverse (red) orientation of the sequence. The nucleotide scale indicated by numbers.

*Agrobacterium tumefaciens* (Smith and Townsend, 1907) Conn, 1942 [=*Rhizobium radiobacter* (Beijerinck and van Delden, 1902) Young et al., 2001] strain C58C1, carrying the binary vector pBI121 [12] and helper plasmid pM90, was used for genetic transformation of Berlin poplar. The T-DNA region of the pBI121 plasmid contains the *nptII* selective and *uidA* reporter gene under the control of the *nos* and CaMV 35S constitutive promoters respectively (Figure 2).

**Figure 3:**
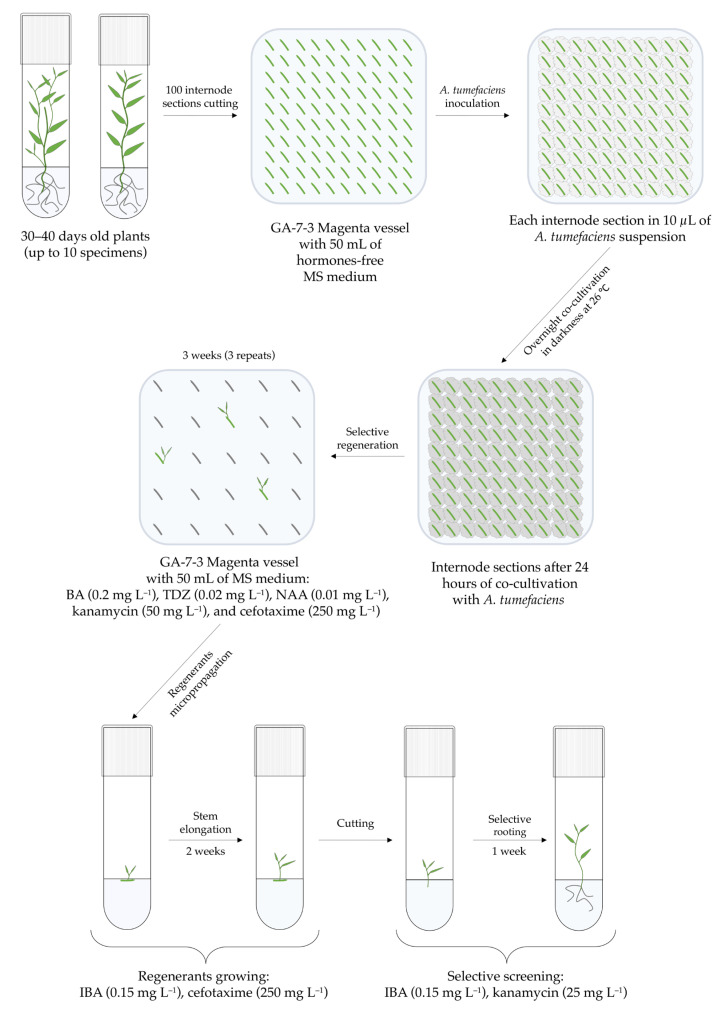
Twelve-week scheme for *Agrobacterium*-mediated transformation of *Populus × berolinensis*.

The *nptII* gene encodes an aminoglycoside 3′-phosphotransferase enzyme that provides resistance to kanamycin by inactivating it in both bacterial cells and plants that have received it [13]. The well-known effects of kanamycin on non-transfected plants include the disruption of adventitious shoot regeneration and the inhibition of shoot rooting during *in vitro* propagation. This is why it is one of the most widely used selection agents [13,14]. The *uidA* gene encodes the beta-glucuronidase enzyme, which is used in the GUS reporter system. When combined with its substrate 5-bromo-4-chloro-3-indolyl-β-D-glucuronide (X-Gluc), it produces a blue coloration in plants, allowing for the visualization of gene expression [15]. The transformation was carried out using an overnight culture of *A. tumefaciens*, obtained by inoculating bacterial glycerol stock stored at −80 °C into 6 mL of liquid YEB medium supplemented with kanamycin (50 mg·L^−1^) and rifampicin (50 mg·L^−1^), followed by incubation in a shaking incubator at 28 °C. The aliquot of overnight bacterial suspension was centrifuged in a 2 mL microtube at 8000 rpm for 2 min and resuspended in 1.8 mL of fresh YEB medium without antibiotics, reaching an optical density of 1.0–1.1 (λ = 600). Subsequently, 10 µL of the obtained bacterial suspension was pipetted onto each of 100 plant explants, which were internode sections (4–6 mm in length without axillary buds) placed on the surface of 50 mL of co-cultivation solid medium (Table 1) in a Magenta vessel GA-7-3 (Figure 3). The same steps were applied for the control vessel, which had 32 explants cultivated in droplets of sterile YEB medium instead of *Agrobacterium* culture. Both the control and experimental vessels were kept in the dark at 26 °C for 24 h. After co-cultivation, the washing step was skipped, and the explants were transferred to the solid BTN regeneration medium (Table 1) containing BA (0.2 mg·L^−1^), TDZ (0.02 mg·L^−1^), and NAA (0.01 mg·L^−1^), but only by 25 explants per vessel. The BTN medium also contained cefotaxime (250 mg·L^−1^) to suppress and eliminate the *Agrobacterium*, as well as kanamycin (50 mg·L^−1^) as a selective agent. The explants were subcultured to fresh medium three times at three-week intervals until the first shoots appeared. All initial explants that had appeared small shoots were transferred to the elongation medium (Table 1). After two weeks of elongation, the shoots were cut and transferred to the rooting media (Table 1), which was supplemented with the aforementioned antibiotics, but kanamycin concentration was reduced to 25 mg·L^−1^. The regenerants that rooted in this medium were considered as potentially transgenic due to their kanamycin resistance. The transgenesis was further verified by PCR-based screening of selected clones. To confirm the absence of *Agrobacterium* contamination in the obtained transgenic plants, their leaf explants were incubated on Petri dishes with YEB medium without antibiotics in an air incubator at 28 °C right up to the death of plant tissue. *Agrobacterium* growth absence indicated no bacterial contamination of the plant tissue. Selected and micropropagated poplar lines were then cultivated on a nutrient medium without antibiotics.

### 2.4. Total DNA Isolation, PCR, and Sequencing

Total DNA was isolated from fresh leaf tissue using the cetyltrimethylammonium bromide (CTAB) method [16], with some authors’ modifications [17].

To prevent possible cross-contamination by DNA left on scissors and forceps used during the poplar micropropagation procedure, sterile disposable plastic pincers were used to sample leaves. To ensure that there was no cross-contamination between different samples during the DNA isolation procedure, the control (non-transfected) plants were handled in parallel. Up to 50 mg of fresh leaf was placed into 2 mL leakage-preventing O-ring screw-cap tubes containing two steel beads (3 and 5 mm diameter) and ground using the automatic homogenizer MiniLys (Bertin Instruments, Montigny-le-Bretonneux, France) at 3000 rpm for 60 s. The obtained plant homogenate was collected on the tube’s bottom through brief centrifugation and diluted in 600 μL of 2% (*m*/*v*) CTAB extraction buffer, with 100 mM Tris-HCl (pH 8.0), 20 mM ethylenediaminetetraacetic acid (EDTA, pH 8.0), 1.4 M NaCl, 3% (*w*/*v*) polyvinylpyrrolidone (PVP-40), and 1% (*v*/*v*) β-mercaptoethanol, mixed well and incubated in a temperature-controlled shaker at 60 °C for 1 h with stirring at 400 rpm. Then, the tubes were briefly spun down, 500 μL of the precooled to 4 °C chloroform–isoamyl alcohol mixture at the ratio of 24:1 (*v*/*v*) was added to the homogenate, and the samples were manually mixed well and centrifuged at 14,000× *g* for 5 min at 4 °C. The upper aqueous phase was transferred to a new tube, an equal volume of the aforementioned chloroform–isoamyl alcohol mixture was added, and the mixture was centrifuged as described above. The upper phase was transferred to the new tube and treated with 40 μg of RNase A (Thermo Fisher Scientific, Vilnius, Lithuania) for 30 min at 37 °C, and DNA was then precipitated by the addition of 0.8 volume of ice-cold (−20 °C) isopropanol to 1 volume of the water phase, followed by incubation for at least 1 h at −20 °C. A DNA pellet was obtained by centrifugation at 14,000× *g* for 15 min at 4 °C, washed twice with 70% ethanol, air-dried at room temperature in the laminar flow hood, resuspended in 100 μL of nuclease-free water preheated at 60 °C, and stored at −20 °C. Obtained DNA were diluted 10 times and used as a template for PCR.

The PCR-based screening of the protentional transformants was conducted using the gene-specific (*uidA* and *nptII*) primers and vector-specific primers flanking the *uidA* gene (Table 2, Figure 2). The PCR was performed using GoTaq G2 Flexi DNA Polymerase (Promega, Madison, WI, USA) in a reaction mixture of 20 μL, containing 1× Green GoTaq Flexi Buffer, 1 unit of GoTaq polymerase, and final concentrations of 2.5 mM of MgCl_2_, 250 µM of each dNTP, and 250 nM of each primer. The amplification conditions for both markers and primer pairs were 96 °C for 5 min; 35 cycles at 96 °C for 20 s; 55 °C for 20 s; and 72 °C for 30 sec, 1 min, or 2 min 30 sec depending on primers pair (Table 2); and a final elongation for 5 min at 72 °C.

Amplicons were visualized in a 1% agarose gel stained with ethidium bromide after electrophoresis, and their sizes (in bp) were compared with the estimated length. For additional verification, the target amplicons with expected size were purified from the gel using the GeneJET Gel Extraction Kit (Thermo Fisher Scientific, Vilnius, Lithuania) and then sequenced in both forward and reverse directions following the Sanger method using the BigDye Terminator Cycle Sequencing Kit version 3.1 (Applied Biosystems, Waltham, MA, USA) and a 3500 Genetic Analyzer (Applied Biosystems and Hitachi, Tokyo, Japan). The same primers applied for PCR were used for sequencing. Raw sequencing data were edited using SnapGene Viewer software version 2.6.2 (GSL Biotech, San Diego, CA, USA). The correspondence of the nucleotide sequence of amplicons to target genes was confirmed using the Nucleotide BLAST online tool on the NCBI platform (www.ncbi.nlm.nih.gov, accessed on 10 November 2023).

### 2.5. GUS Staining Assay

All selected transgenic plants were examined for constitutive *uidA* expression using the histochemical staining method described in Tzfira et al. [18] with some modifications. Two-month-old *in vitro* transgenic poplars were removed from the test tubes and rinsed with distilled water to remove the nutrient media from the roots. Each intact plant was placed in a 50 mL plastic syringe filled with 0.2 M sodium phosphate buffer (pH 7.0) containing 0.025% X-Gluc (preliminarily dissolved in DMSO), 10 mM EDTA, and 1% (*v*/*v*) Triton X-100. The plants were then exposed to vacuum infiltration for 1 min, applied by manual piston movement. To detect the GUS enzymatic activity, the plants were transferred to the glass test tubes and incubated in the same X-Gluc buffer at 37 °C for 24 h until the plant tissues turned blue. The developed coloration in whole plants was captured using a photo camera.

## 3. Results

### 3.1. PCR-Based Screening of Kanamycin-Selected Regenerated Poplar Lines

**Figure 4:**
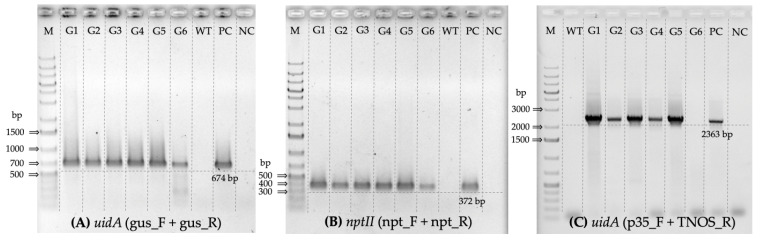
Results of PCR-based screening for six kanamycin-selected *Populus × berolinensis* regenerated lines (G1–G6) using primers: (**A**) specific to the inner part of the *uidA* gene, (**B**) specific to the inner part of the *nptII* gene, and (**C**) specific to the 35S promoter and NOS terminator, flanking the *uidA* gene. M—GeneRuler 1 kb Plus DNA Ladder size standard (Thermo Fisher Scientific, Vilnius, Lithuania); WT—the control line of poplar (wild type); PC—*uidA*/pBI121 plasmid DNA (positive control); and NC—no DNA template control (negative control).

After plant regeneration on BTN-medium in the presence of kanamycin, six lines (referred to as G1-G6 below) of potential transformants were selected. All selected plants also showed efficient rooting in the kanamycin-supplementing medium. PCR analysis using gene-specific primers revealed that all lines contained both *uidA* and *nptII* genes (Figure 4A,B). Moreover, the following analysis of the obtained amplicon sequences using BLAST revealed 100% identity with the *uidA* and *nptII* genes, which were part of the pBI121 complete sequence deposited in GenBank (ID: AF485783.1). Consequently, all six lines of the selected regenerants could be considered transgenic.

Additionally, PCR was performed using primers which were specific to the 35S promoter and NOS terminator flanking *uidA* from both sides to confirm the insertion of the full-length reporter gene. The results only showed a positive signal for the G1–G5 lines but not for the G6 (Figure 4C).

### 3.2. Verification of nptII Expression in Transgenic Poplar Lines

**Figure 5:**
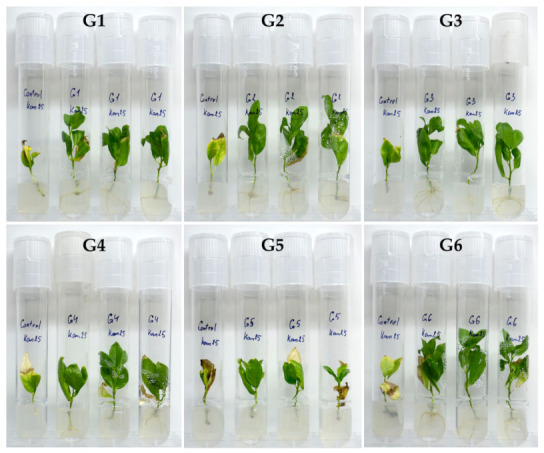
Rooting of *Populus × berolinensis* plants on medium with kanamycin concentration of 25 mg·L^−1^ (refer to Table 2). G1–G6—the transgenic lines; Control—the non-transgenic line (wild type).

The maintenance of *nptII* gene expressions, subsequent to their transfection into the poplar genome as part of T-DNA, was confirmed by the successful shoot rooting and the second-round regeneration in the presence of kanamycin. In particular, the shoot rooting during *in vitro* micropropagation of all six transgenic lines was nearly 100% while no rooting was observed in the control plants (Figure 5).

**Figure 6:**
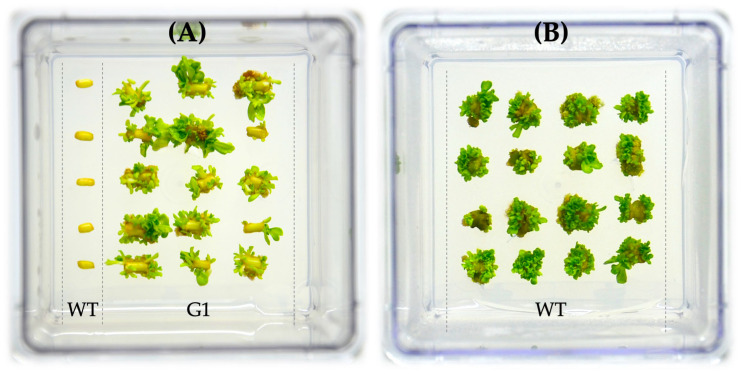
Regeneration of *Populus × berolinensis* on a BTN-medium (Table 2) with 75 mg·L^−1^ of kanamycin (**A**) and without kanamycin (**B**). G1—transgenic line; WT—control line (wild type).

Furthermore, the transgenic lines exhibited successful second-round regeneration on the BTN-medium (Table 2) in the presence of kanamycin, but at an elevated concentration, which was 75 mg·L^−1^ (Figure 6).

The figure depicts the regeneration of the G1 line on the BTN-medium with kanamycin (Figure 6A), and a similar outcome was observed for the other five transgenic lines. The plant regeneration on the control (non-transgenic) explants was blocked in case of the kanamycin presence (Figure 6A). Simultaneously, the regeneration on the kanamycin-free medium was effective for control explants (Figure 6B).

### 3.3. Verification of uidA Expression in Transgenic Poplar Lines

**Figure 7:**
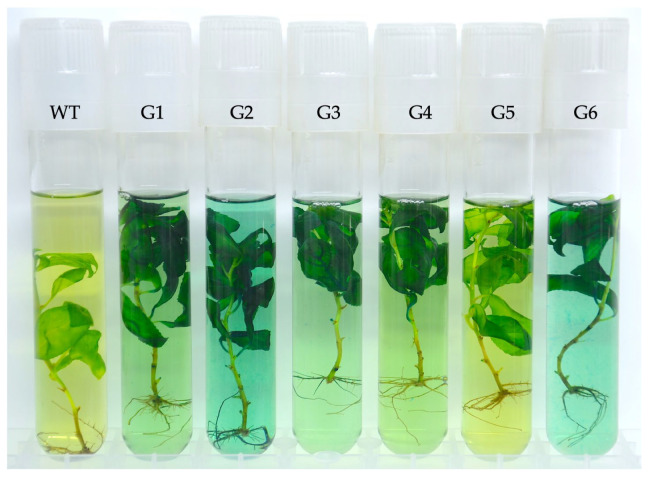
Results of GUS staining assay of *Populus × berolinensis* plants. G1–G6—transgenic lines; WT—control line (wild type).

To assess *uidA* expression, we utilized the GUS reporter system. Specifically, all six transgenic lines exhibited blue coloration after 24 h of exposure to X-Gluc, while no color changes were observed in the control group (Figure 7).

While our primary objective was not to pinpoint the specific tissues expressing GUS, we observed that the blue coloration in transgenic plants was predominantly present in the majority of the leaf blades and also discernible in the stems and roots. In most instances, the incubation buffer consistently displayed intense coloration.

## 4. Discussion

Following our protocol, we successfully obtained six transfected lines of *Populus × berolinensis* through *Agrobacterium*-mediated genetic transformation. The transformation efficiency was found to be 6% considering the 100 explants originally taken. The efficiency of the *Agrobacterium*-mediated transformation of poplar can significantly vary, ranging from 0.4% to 75%, depending on various factors such as the construct being inserted, explant type, co-cultivation time and temperature, application of acetosyringone, types and content of regenerating hormones, and the genotype used [9,19,20,21,22]. A high efficiency (more than 50%) of *Agrobacterium*-mediated transformation generally achieved by using a callus, which provides a higher amount of target cells compared to plant organ segments, thereby increasing the chance of a successful transformation. Our simplified protocol showed a 6% transformation efficiency that is lower than average but still acceptable when compared to the lowest efficiencies obtained in different studies, especially considering the simplicity of the developed protocol. 

All transgenic clones selected in our study exhibited a positive PCR signal for both the selective and reporter genes, and their expression was maintained following their transfection into the poplar genome as part of the T-DNA. However, the G6 line was the only one that did not show a positive signal of PCR prepared with primers specific to the 35S promoter and NOS terminator which flanked the *uidA* gene. The absence of a signal in the G6 clone may indicate the transfer of the non-full-length *uidA* gene or the omission of the partial or full NOS terminator sequence due to its close location to the left border of T-DNA. Nevertheless, the GUS enzymatic activity remained in the G6 line, which may indicate the integrity of the main part of the coding sequence. Additionally, all transgenic lines effectively rooted and regenerated on the medium containing kanamycin. The control lines of poplar also effectively regenerated on the kanamycin-free BTN-medium. Hence, that used BTN-medium (Table 2) can be effectively applied for the micropropagation of both control and transgenic poplars.

The presented protocol of *Agrobacterium*-mediated genetic transformation has several advantages compared to those previously described in other studies some of which are described below.

**Co-cultivation**. The most commonly used co-cultivation technique involves incubating plant explants in a liquid *Agrobacteria* suspension and liquid media for hours or even several days. This is often accompanied by continuous shaking, followed by several steps of rinsing and drying the explant over sterile paper before the nutrient media change [4,23,24]. However, this technique has several disadvantages, such as the complicated handling of the explants during their transfer from liquid medium to a solid, and the stressful conditions due to their extensive handling, which can lead to wounded plant tissues. The alternative technique we present involves incubating the explants in small droplets of bacterial suspension directly on the surface of a solid agar-based medium for co-cultivation. Subsequently, the explants are transferred to the regeneration medium, omitting the potentially damaging washing step. This approach facilitates the transfer of the explants and maintains them in relatively stable and safe conditions.

**Regeneration medium.** We have demonstrated that a BTN-medium can be applied throughout the entire regeneration process without altering the concentration or composition of hormones. Our approach simplifies the *Agrobacterium*-mediated transformation of poplar and reduces costs associated with the process. An additional feature of the used BTN-medium is that it employs a combination of two cytokinins, BA and TDZ, both in low concentrations. Cytokinins (including BA) can be biologically inactive and after being taken up by plant tissue, only a very low percentage remains in free unconjugated form. TDZ is an exception and is conjugated only at a very low rate, whereas most purine-type cytokinins are considered to be somewhat chemically unstable [18,25]. Thus, the application of TDZ in the medium reduces the need for multiple transplanting explants during regeneration. It should be noted that the simultaneous use of these two cytokinins was also applied for poplar regeneration in different studies; however, in different concentrations to those used by us [8,26].

**Acetosyringone.** Acetosyringone, a phenolic compound produced from plant wounds, is known to induce the activation and expression of virulence (*vir*) genes in *Agrobacterium* which are responsible for transferring T-DNA into a plant genome [27]. Various studies have shown that the addition of acetosyringone to the plant transformation protocol improves its efficiency for different plants [8,28], including poplar [4,9,22,26]. However, its use at different concentrations may not always have a significant effect or may only slightly increase the transformation efficiency, typically not more than 2.5–3 times higher than in the control. Additionally, the application of acetosyringone significantly increases the costs, time, and effort required for the procedure. Our study confirms that transgenic poplar plants can be effectively obtained even without the addition of acetosyringone to the transformation protocol, presenting a cost- and time-effective alternative, particularly if obtaining a high number of different transfected lines is not the key priority.

**Direct morphogenesis.** During the genetic transformation process, the *in vitro* regeneration of plants from a callus can result in chimerism in the selected plants [10,29]. Therefore, selecting transformants through direct morphogenesis on the plant explant appears to be a more efficient approach for genetic transformation, as it reduces the likelihood of chimerism occurring.

**Explant types.** In our study, we employed internode sections from the long-term *in vitro* culture of Berlin poplar as explants. While leaf cuts are frequently used for regeneration and transformation [3,22,23,26] due to their accessibility, they have limitations when used *in vitro*. The thin and delicate nature of leaves can result in issues with further handling and regeneration due to cell stress or even death. Prolonged exposure of leaf tissue to air for slicing can cause rapid drying, leading to additional challenges in the regeneration process. Other explant types, such as leaf petioles, root segments, and calli [9,24], are also widely used, each with its own advantages and disadvantages. Although internode sections of shoots are sometimes used in studies, they are not as commonly used as leaves [4,8,30]. Many studies prefer to use freely growing plants as a tissue donor for regeneration, rather than *in vitro* cultures [4,22]. This approach has the advantage of not requiring long-term maintenance of *in vitro* plant cultures, but it increases the risk of bacterial or moldy contamination of nutrient media and infection of regenerants. In our opinion, stems (internode sections) are more suitable than other types of *in vitro* explants for shoot regeneration in poplar.

## 5. Conclusions

As a result of our study, we have developed and applied a protocol for the genetic transformation of Berlin poplar, including transgenic shoot regeneration with the following key particularities.

1. Solid medium co-cultivation step. Explants were co-cultivated in 10 µL droplets of *Agrobacterium* suspension directly on the surface of a solid agar-based medium without subsequent rinsing or sterile paper drying after inoculation.
2. Nutrient medium. A only one regeneration MS-based medium supplemented with BA (0.2 mg·L^−1^), TDZ (0.02 mg·L^−1^) and NAA (0.01 mg·L^−1^) was used.
3. Explant type and direct morphogenesis. *In vitro* internode sections without axillary buds were used as explants for direct morphogenesis.
4. Acetosyringone absence. Acetosyringone was not used as an induction agent for the *vir* genes during the genetic transformation.

The transformation efficiency, obtained by applying the developed protocol and considering the 100 explants taken originally, was 6%.

## Figures and Tables

**Table 1:** Nutrient media for *Populus × berolinensis* micropropagation and selective regeneration.

Components	Co-Cultivation	Regeneration (BTN-Medium)	Elongation	Rooting **
MS5524 *	4.3 g·L^−1^	4.3 g·L^−1^	4.3 g·L^−1^	4.3 g·L^−1^
Thiamine	1 mg·L^−1^	1 mg·L^−1^	1 mg·L^−1^	1 mg·L^−1^
Pyridoxine	0.5 mg·L^−1^	0.5 mg·L^−1^	0.5 mg·L^−1^	0.5 mg·L^−1^
Nicotinic acid	0.5 mg·L^−1^	0.5 mg·L^−1^	0.5 mg·L^−1^	0.5 mg·L^−1^
Meso-Inositol	50 mg·L^−1^	50 mg·L^−1^	50 mg·L^−1^	50 mg·L^−1^
Sucrose	3 g·L^−1^	3 g·L^−1^	2 g·L^−1^	2 g·L^−1^
Agar	7 g·L^−1^	7 g·L^−1^	7 g·L^−1^	7 g·L^−1^
6-Benzylaminopurine (BA)	–	0.2 mg·L^−1^	–	–
Thidiazuron (TDZ)	–	0.02 mg·L^−1^	–	–
1-Naphthaleneacetic acid (NAA)	–	0.01 mg·L^−1^	–	–
Indole-3-butyric acid (IBA)	–	–	0.15 mg·L^−1^	0.15 mg·L^−1^
Kanamycin	–	50 mg·L^−1^	50 mg·L^−1^	25 mg·L^−1^
Cefotaxime	–	250 mg·L^−1^	250 mg·L^−1^	–
pH	5.7	5.7	5.7	5.7

* Murashige and Skoog Basal Salt Mixture (Sigma–Aldrich, Germany). ** A medium without kanamycin was also used for the routine *in vitro* cultivation of poplar.

**Table 2:** The primers used for detecting the reporter (*uidA*) and selective (*nptII*) genes in transgenic poplar.

Primer Name	Target	Sequence (5′ –> 3′)	Ta, °C	Amplicon, bp
npt_F	*nptII*	TGGAGAGGCTATTCGGCTATGA	55	372
npt_R		GATGTTTCGCTTGGTGGTCG		
gus_F	*uidA*	CAACGAACTGAACTGGCAG	55	674
gus_R		AGAGGTTAAAGCCGACAGC		
p35_F	35S promoter (*uidA*)	CCATTGCCCAGCTATCTGTCACT	55	2363
TNOS_R	NOS terminator (*uidA*)	CCCATCTCATAAATAACGTCATGCA		

## Data Availability

Data are contained within the article.
